# Dendrobium huoshanense polysaccharide inhibits NSCLC proliferation and immune evasion via FXR1-IL-35 axis signaling pathway

**DOI:** 10.1007/s11418-025-01894-7

**Published:** 2025-04-21

**Authors:** Xinying Zhu, Guoquan Yin, Jiaqian Xu, Xiaolei Tang, Fangliu Yu

**Affiliations:** 1 Translational Medicine Center, The Second Affiliated Hospital of Wannan Medical College, Wuhu, 241000 Anhui Province China; 2 Clinical Laboratory, Yangzhou Blood Center in Jiangsu Province, Yangzhou, 225007 Jiangsu Province China; 3 Department of Medical Microbiology and Immunology, School of Preclinical Medicine, Wannan Medical College, Wuhu, 241001 Anhui Province China

**Keywords:** Tumor microenvironment, IL-35, iTr35, Immune evasion

## Abstract

Dendrobium huoshanense has received special attention for its advantages in the treatment of lung cancer, but the underlying molecular mechanisms are not yet well understood. First, we obtained 8 active ingredients and 159 effective action targets of Dendrobium huoshanense using network pharmacology, and searching target interactions through STRING, constructing the PPI network and KEGG, GO and Hallmark enrichment analysis. Then, we combined target’s enrichment analysis and GSEA enrichment analysis of IL-35, indicating the mechanism of cDHPs for non-small cell lung cancer (NSCLC) may be related to tight junction and NSCLC pathway. Further, FXR1 and ACTR3 were identified as core therapeutic targets, and high expression of FXR1 or ACTR3 was significantly associated with poor prognosis of patients. The analysis of single-cell data also indicated that the percentage of CD4-CTLA4-Treg cells may be increased by the expression of IL-35, resulting in a suppressive immune microenvironment. Next, In vivo experiment, we detected iTr35 by flow cytometry, detected IL-35 level by RT-PCR, Western blotting and ELISA, and detected NK cell activity to explore the immunomodulatory effects and anti-tumor mechanism of cDHPs. After cDHPs administration, the conversion of CD4^+^ T cells to iTr35 is inhibited, p35 and EBI3 in both protein and mRNA levels, the levels of IL-35 and IL-4 in serum decreased. The levels of IFN-γ, while the activity of NK cells in mice increased, enhancing the anti-tumor immune effect of the organism. Finally, analysis of sequencing data from the immunotherapy cohort of tumor-bearing mice obtained from the TISMO database shows that the combination of cDHPs and PD-1/PD-L1 antibodies improves effector and thus PD-1/PD-L1 antibody efficacy. These findings suggest that cDHPs inhibit NSCLC proliferation and immune escape via the FXR1-IL-35 axis signaling pathway.

**Graphical abstract:**

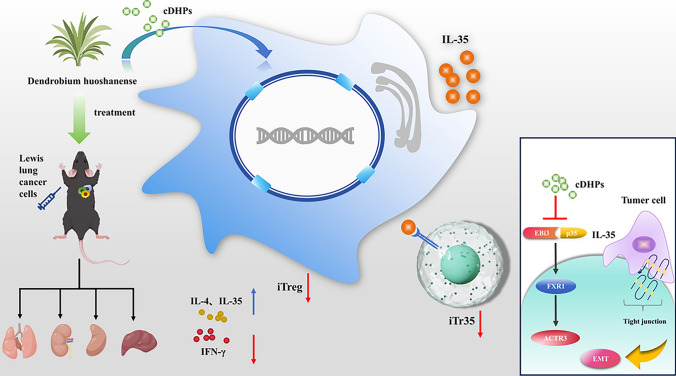

**Supplementary Information:**

The online version contains supplementary material available at 10.1007/s11418-025-01894-7.

## Introduction

Lung cancer is one of the common causes of cancer-related deaths globally, responsible for almost 2.5 million new cases (12.4% of all cancers globally) in 2022, and estimated 1.8 million people die from lung cancer, followed by colorectal cancer, liver cancer, female breast cancer and stomach cancer [1]. Chemotherapy, video-assisted thoracoscopic and stereotactic ablative body radiotherapy are all options for treating lung cancer [2]. The significant side effects, propensity for cancer cell resistance, and danger of recurrence associated with today’s chemotherapeutic agents provide additional difficulties for cancer patients[3]. In recent years, oncology patients have reason to hope thanks to the advancement of immunotherapy for cancers, particularly immune checkpoint inhibitor (ICI) therapies like anti-programmed cell death-1 (anti-PD-1) and programmed death-ligand 1 (PD-L1), which have the greatest potential for application. This results in an immunosuppressive tumor microenvironment, or ‘cold’ tumor. Therefore, there has been a lot of interest in the development of new medications that stimulate anti-tumor immunity and alter the tumor microenvironment.

Nevertheless, tumors frequently control anti-tumor immunity by enlisting tumor-associated cells, particularly T regulatory cells (Tregs) and M1 macrophages, to stave off immune attacks [4]. To maintain self-tolerance, prevent autoimmune disorders, and limit chronic inflammation, the body uses CD4^+^CD25^+^Foxp3^−^ Tregs primarily [5]. Over the past 40 years, cytokines and cytokine receptors have been extensively explored as cancer targets or cancer therapies. A heterodimeric pair comprising EBI3 and IL-12A (p35) makes up the inhibitory cytokine interleukin-35 (IL-35), a recently discovered member of the IL-12 family. It has been demonstrated that IL-35 induces the conversion of conventional T cells (Tconv) into induced regulatory T cells (iTregs) and contributes to immune regulation by promoting IL-35 expression through a positive feedback loop. Conversely, natural regulatory T cells (nTregs) produce IL-35 to induce the conversion of CD4^+^Foxp3^−^T cells into iTregs [6].

An increasing number of research have revealed that using herbal medication has some distinct benefits. Dendrobium huoshanense C.Z. Tang and S.J. Cheng is a perennial herb of the genus Dendrobium in the family Orchidaceae. The primary active component of Dendrobium huoshanense is the Dendrobium huoshanense polysaccharide(cDHPs), which is mostly made up of more than 10 monosaccharides such mannose and glucose. cDHPs has been proven to have anti-inflammatory [7], anti-oxidant [8], anti-viral [9], anti-tumor [10], hepatoprotective, and immunity-activating properties [11]. However, the anti-tumor effects of cDHPs lack in-depth research, and the specific mechanism of anti-tumor immunomodulatory effects is also lacking. In this study, we found that cDHPs inhibited the growth of lung adenocarcinoma (LUAD) transplants in mice and prevented the conversion of CD4^+^ T cells into IL-35-producing induced regulatory T cells (iTr35) to activate anti-tumor immunity by inhibiting the FXR1-IL-35 axis.

## Materials and methods

### Screen the active ingredients and targets of dendrobium huoshanense

We obtained the active components of Dendrobium huoshanense and their targets of action from the database via cancer HSP [12] (https://old.tcmsp-e.com/CancerHSP.php) and Symmap [13] (https://www.symmap.org). Their targets for the components were further predicted by SwissTargetPrediction [14] (http://swisstargetprediction.ch) based on their chemical structures. The active ingredients were screened based on drug-like properties (DL) ≥ 0.18 and oral bioavailability (OB) ≥ 30%, and then we translated the target names of these active ingredients into gene names via the Uniprot database (https://www.uniprot.org), and finally we used Cytoscape 3.9.1 software to construct a Dendrobium huoshanense active ingredients and the relationship network between the target genes.

### The analysis of PPI network, KEGG, GO, and Hallmark

We analyzed the interactions between the potential therapeutic targets of Dendrobium huoshanense through the STRING database (https://cn.string-db.org) and used Cytoscape software to construct a protein–protein interaction (PPI) network. To further investigate the biological functions of the targets of Dendrobium huoshanense and to explore the possible molecular mechanisms of Dendrobium huoshanens, we used R4.0.4 software and Metascape (https://metascape.org) for Gene Ontology (GO), Kyoto Encyclopedia of Genes and Genomes (KEGG), and Hallmark enrichment analysis.

### Study cohort and expression analysis

A cohort of LUAD patients from The Cancer Genome Atlas (TCGA), which contains RNA sequencing data and phenotypic information from 576 LUAD patients, was included in this study. We used GEPIA2 (http://gepia2.cancer-pku.cn) for differential expression analysis and survival analysis of four genes, Fragile X-related protein-1 (FXR1), actin-related protein 3 (ACTR3/ARP3), altered tubulin alpha 8 (TUBA8) and nuclear factor kappa-B subunit 2 (NF-κB2).

### GSEA

We performed GSEA (GSEA V4.2.3) enrichment analysis on RNA-seq data from 576 LUAD patients from TCGA. The samples were classified as high-expression (> 50%) or low-expression (< 50%) based on the level of IL-35 expression. The c2.cp.kegg.v7.4.symbols.gmt gene set was selected for GSEA analysis of the above genes separately. Based on gene expression profiles and phenotypic groupings, a minimum gene set of 5 and a maximum gene set of 5000 were set with 1000 resampling, and a *P* value of < 0.05 and an FDR of < 0.25 were considered statistically significant.

### Single-cell RNA-seq data and analysis

We obtained single-cell transcriptome sequencing data for two sets of LUAD, GSE139555 and GSE131907, from the GEO database and analyzed them using the scTIME Portal (http://sctime.sklehabc.com/unicellular/home).

### Chemicals

cDHPs was purchased from the Sichuan Weikeqi Biological Technology Co., Ltd. (WKQ-0027085, Chengdu, China) with a purity > 90% tested by high-performance liquid chromatograph.

### Cell line, tumor models and treatment

Lewis lung carcinoma (LLC) was purchased from ATCC and cultured in Dulbecco's modified Eagle medium (DMEM) containing 10% fetal bovine serum (FBS), 100 units/mL of penicillin and 100 µg/mL of streptomycin at 37 °C in a humidified incubator with 5% CO_2_. Routine testing of LLC confirmed the absence of mycoplasma contamination, and limited generation cultures were performed.

Thirty C57BL/6 female mice, 3 weeks old, 15–17 g, were purchased from Changzhou Cavens Laboratory Animal Co., LTD. (Changzhou, China). The animals were housed and maintained under optimal conditions of light, temperature, and humidity, with free access to food and water. To create an LLC model, 1 × 10^6^ LLC was resuspended in 100 µL PBS and inoculated under the skin in the right axilla of mice after 1 week of adaptive feeding. Thirty mice were randomly divided into 3 groups (*n* = 10): normal, model, and cDHPs groups. Three days after inoculation, 200 mg/kg cDHPs were given to the cDHPs group and equal amounts of distilled water were given to the normal and model groups by daily gavage. All experimental procedures were authorized by the Wannan Medical College’s Animal Experimentation Ethics Committee and followed the “Principles of Laboratory Animal Care”. After 21 days of treatment, the mice were euthanized. Single-cell suspensions of primary cell isolates were prepared from spleens using a mechanical grinding method. Spleen and blood specimens were taken, ground, and then centrifuged and suspended in 1640 medium using red cell lysate. Flow cytometry was used to detect iTr35, CD4^+^ Foxp3^-^Tconv, and lung tissue and remaining tumor tissue were stored in 4% paraformaldehyde or ˗ 80 °C in a refrigerator to measure other biochemical parameters.

### Flow cytometry assay

Antibodies used for flow cytometry analysis of CD4^+^Foxp3^- ^Tconv, iTr35, were sorted using a BD flow cytometer as follows: PerCP-Cy 5.5 Rat Anti-Mouse Foxp3 (563,902, BD Bioscience, USA), FITC Rat Anti-Mouse CD4 (553046, BD Bioscience, USA), Human/Mouse IL-12/IL-35 p35 PE-conjugated Antibody (IC2191P, R&D System, USA), Mouse IL-27/IL-35 EBI3 Subunit APC-conjugated Antibody (IC18341A, R&D System, USA), CD16/CD32 Monoclonal Antibody(93) (14-0161-81, Invitrogen, USA), Cell Stimulation Cocktail (500 ×) (00-4970, Invitrogen, USA), Protein Transport Inhibitor Cocktail (500 ×) (00–4980, Invitrogen, USA), Transcription Factor Buffer Set (51-9008100, BD Biosciences, USA).

### Quantitative real-time PCR (RT-PCR)

Total RNA was extracted from spleens using TRIzol (15596026, Invitrogen, USA), reverse transcribed by HyperScript III RT SuperMix for qPCR with gDNA Remover (R202, NovaBio, China) and PCR amplified using NovoStart SYBR qPCR SuperMix Plus (E096, novoprotein, China). PCR products were amplified using the following thermal cycling: 95 °C for 1 min, 95 °C for 20 s, 60 °C for 1 min, 40 cycles, using 2% agarose containing ethidium bromide The PCR amplification products were observed on gels, and parameters were used for amplification data analysis using the ΔΔCt method. Primers were designed using Primer Premier 5 software. The primers used are listed in Table 1.

**Table 1:** Primers used for RT-PCR amplifications

Gene	Primer (5′ > 3′)
p35	Forward: CAATCACGCTACCTCCTCTTTT
	Reverse: CAGCAGTGCAGGAATAATGTTTC
EBI3	Forward: CATTGCCACTTACAGGCTCG
	Reverse: TGCAGTGACATTTAGCATGTAGG
β-actin	Forward: GGCTGTATTCCCCTCCATCG
	Reverse: CCAGTTGGTAACAATGCCATGT

### Western blotting

Spleen tissue (50 mg) was ground and lysed with 500 μL of RIPA lysate (P0013B, Beyotime, China). The resulting cell lysates were centrifuged at 12,000 rpm for 15 min at 4 °C. The BCA kit (P0012S, Beyotime, China) was used to detect protein expression in the lysate, and equal amounts of protein were separated by SDS-PAGE and transferred to PVDF membranes (IPVH00010, Millipore, USA), and the membranes were closed with 5% skimmed milk powder before the addition of primary antibody (1:1000). The membrane was incubated overnight at 4 °C and then washed with 0.1% Tween-20 in tris-buffered saline. Secondary antibodies were mixed with HRP (1:2000) and incubated at 37 °C for 1 h. Protein expression was determined using the ECL kit and chemiluminescent image detection system and quantified using ImageJ software. In the western blot analysis, the following antibodies were used: IL-12A rabbit mAb (A20383, ABclonal, China), EBI3 rabbit mAb (A19613, ABclonal, China), and β-actin antibody (#4970, CST, USA).

### Enzyme-linked immunosorbent assay (ELISA)

Following serum collection, ELISA kits were used to detect the concentrations of IFN-γ (CSB-E04578m, CUSABIO, China), IL-35 (CSB-E13145m, CUSABIO, China), and IL-4 (CSB-E04634m, CUSABIO, China).

### Natural killer (NK) cell activity assay

The spleen tissue was ground, and NK cells were extracted after lysing the red blood cells. The target cells of NK cells were co-cultured with NK cells at 100:1, 50:1, 25:1, and the killing ability of NK cells was detected by adding Cell Counting Kit-8 (BS350B, Biosharp, China) after 20 h.

### Statistical analysis

Statistical analysis was performed using Student's t-test for comparison of two groups or one-way analysis of variance for comparison of more than two groups followed by Tukey's multiple comparison test. For multiple testing, a Bonferroni post hoc test of p values was done. Statistical calculations were performed using GraphPad Prism (GraphPad, San Diego, USA). The data were expressed as the means standard deviations of at least three independent experiments. *p* value < 0.05 was considered statistically significant.

## Result

### Active ingredients and targets of dendrobium huoshanense, PPI network analysis

**Fig. 1:**
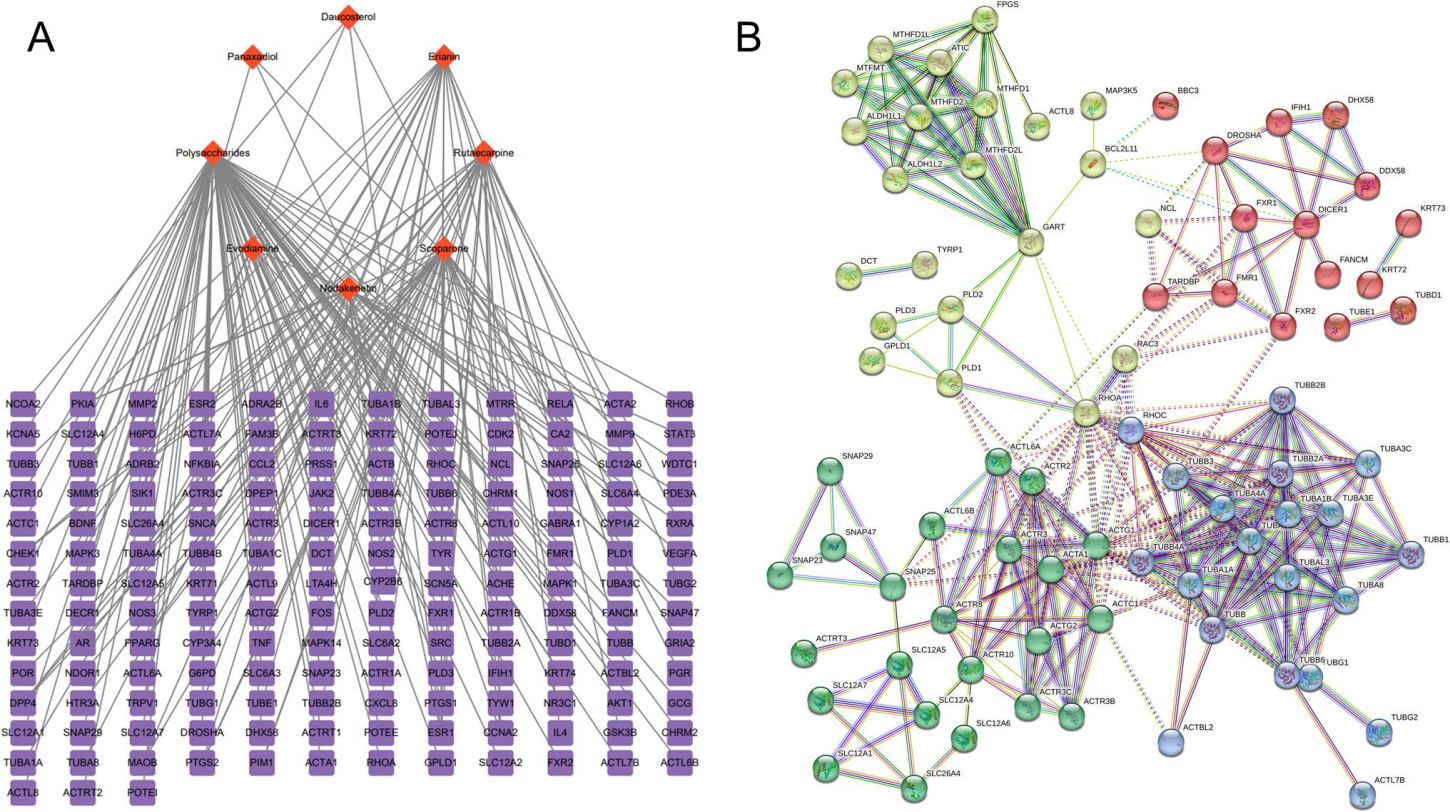
Active ingredients and targets of dendrobium huoshanense, PPI network analysis. **A** The network of the relationship between the active ingredients and the targets of Dendrobium huoshanense. **B** The PPI network of Dendrobium huoshanense’s targets

The active ingredients of Dendrobium huoshanense were analyzed and the targets were predicted, 8 active ingredients (Polysaccharides, Daucosterol, Scoparone Rutaecarpine, Nodakenetin, Panaxadiol, Evodiamine) and 159 targets of Dendrobium huoshanense were obtained (Fig. 1A, Supplementary material 3). Figure 1B depicts the PPI network relationship between the active targets of Dendrobium huoshanense (Supplementary material 1).The K means clustering (*K* = 4) demonstrates that FXR1, DICER1, ACTR3, GART, and TUBA1B are more connected in their respective clusters.

### Results of KEGG, HALLMARK and GO enrichment analysis

**Fig. 2:**
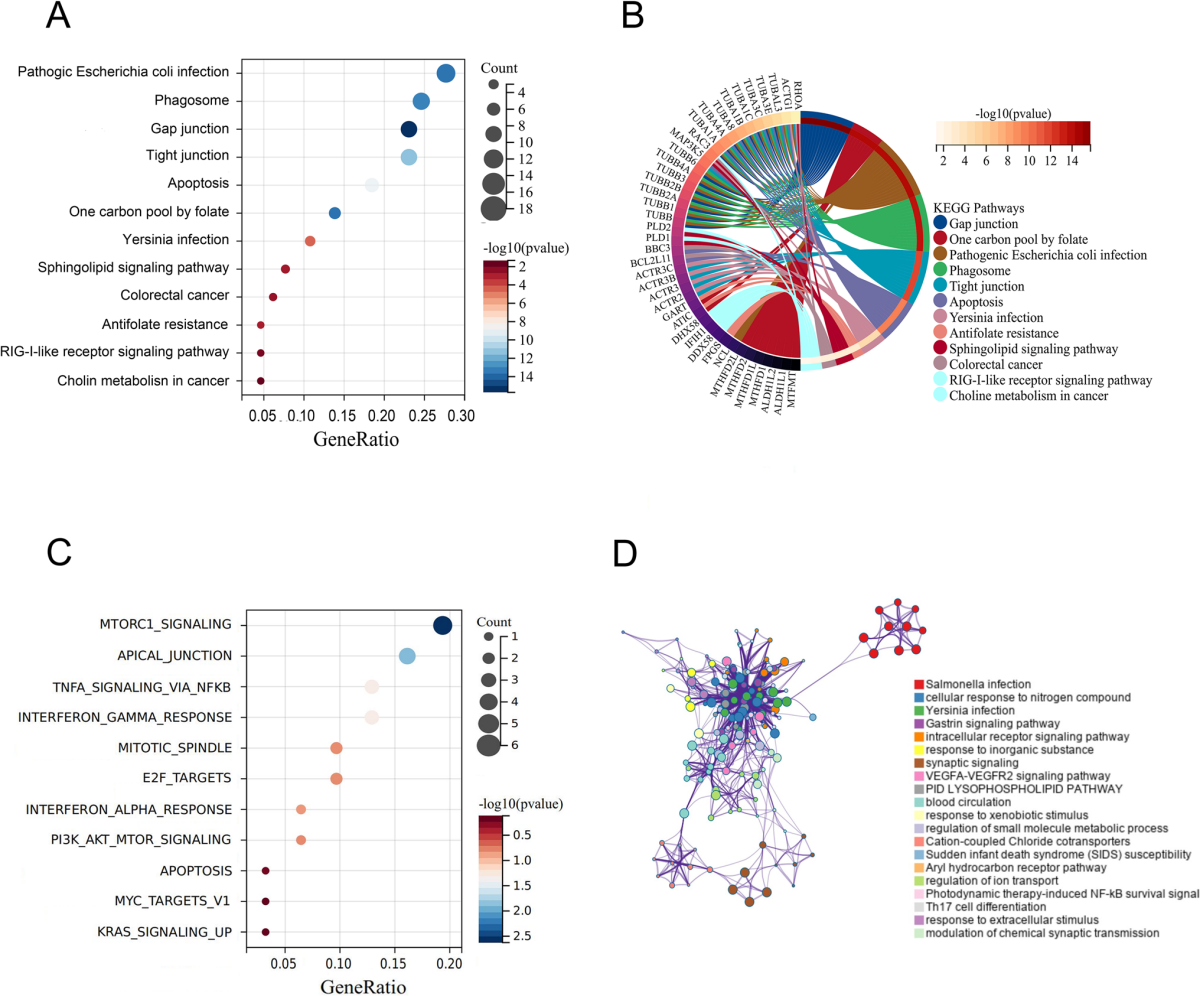
Results of KEGG, HALLMARK and GO enrichment analysis. **A** KEGG enrichment analysis of therapeutic targets (the top 12 results). **B** Chord Diagram of KEGG enrichment analysis. **C** HALLMARK enrichment analysis of therapeutic target. **D** GO enrichment analysis of therapeutic target

To further explore the mechanism and the biological functions of the target genes of Dendrobium huoshanense, we performed KEGG and HALLMARK enrichment analysis of Dendrobium huoshanense targets using R software. In the KEGG enrichment analysis, the drug targets were mainly enriched in pathogenic escherichia coli infection, phagosome, gap junction, tight junction, apoptosis, one carbon pool by folate, colorectal cancer, etc. pathways (Fig. 2A, B). HALLMARK gene set enrichment analysis showed that drug targets were enriched in mTORC1 signaling, apical junction, TNF-α signaling via NF-κB, interferon-γ response, apoptosis, KRAS signaling up, etc. (Fig. 2C). This suggests that the targets of Dendrobium huoshanense are associated with numerous tumor development and tumor immune pathways. As shown in Fig. 2D, GO enrichment analysis.

### Multi-omics analysis reveals that IL-35 is highly expressed in LUAD and is associated with the formation of an immunosuppressive TME

**Fig. 3:**
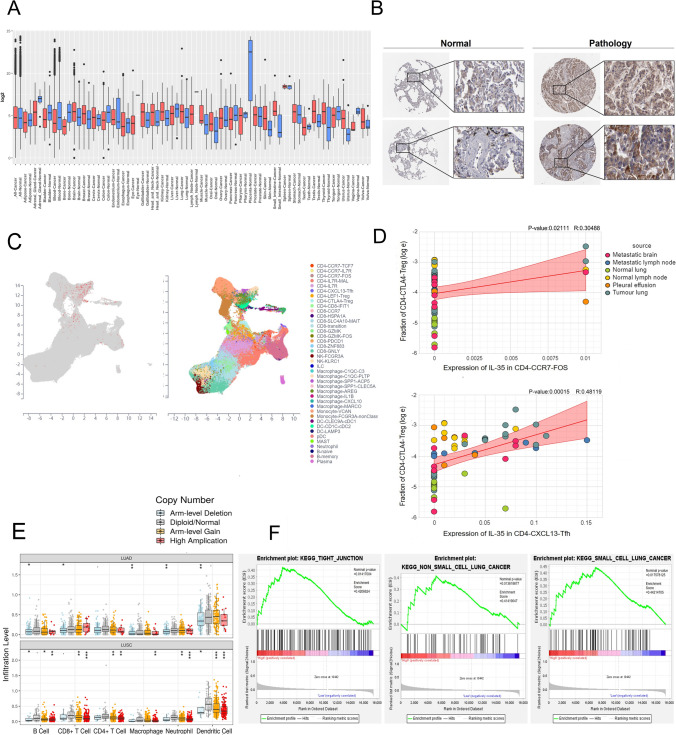
Multi-omics analysis reveals that IL-35 is highly expressed in LUAD and IL-35 regulates the development of NSCLC. **A** Pan-cancer analysis of IL-35. **B** Immunohistochemical analysis of IL-35 in lung tissue from normal subjects and patients with LUAD. **C** IL-35 expression in single-cell sequencing cohort. **D** Expression and cell composition correlation between IL-35 expression in CD4-CCR7-FOS T cell and CD4-CXCL13-Tfh and composition of CD4-CTLA4-Treg. **E** Analysis of immune infiltration abundance of IL-35 in LUAD and LUSC. **F**: The GSEA enrichment analysis of IL-35

Dendrobium huoshanense most likely exerts its anti-cancer properties via inducing anti-tumor immunity, according to our prior study and Fig. 2. Pan-cancer analysis showed that IL-35 was significantly highly expressed in lung cancer (Fig. 3A). We used immunohistochemical results from the HPA database (https://www.proteinatlas.org/). The results also showed that IL-35 expression was elevated in pathological lung cancer tissues compared to normal tissues (Fig. 3B), all of which could indicate that IL-35 is highly expressed in lung cancer. Analysis of single-cell sequencing data also showed that IL-35 was mainly expressed at higher levels in the CD4^+^ T cell population in the tumor microenvironment (Fig. 3C), and analysis by expression and cell composition correlation suggested that CD4-CXCL13-Tfh, CD4-CCR7-FOS cells may increase the immunosuppressive CD4-CTLA4-Treg occupancy by expressing IL-35, resulting in an immunosuppressive immune microenvironment (Fig. 3D). Next, we investigated the correlation between changes in IL-35 copy number and the abundance of immune cells that play a major role in anti-tumor immunity, such as B cells, CD8^+^ T cells, CD4^+^ T cells, macrophage, neutrophil cells, and dendritic cells, in LUAD and lung squamous carcinoma (LUSC), and showed a decrease in immune infiltration compared to normal when the gene encoding IL-35 was amplified, especially in CD8^+^ T cells, CD4^+^ T cells and DC cells (Fig. 3E), suggesting that IL-35 is an important target for immune evasion.

### IL-35 regulates the development of NSCLC through the tight junction pathway and non-small cell lung cancer pathway

To further investigate the role of IL-35 in non-small cell lung cancer (NSCLC), we obtained transcriptional sequencing data from the TCGA database of 576 tumor and normal tissues of LUAD patients, divided the samples into high and low expression groups in the form of median IL-35 expression, and performed GSEA enrichment analysis, which showed that both NSCLC and SCLC pathways were activated in the high expression group (Fig. 3F). This indicates that the activation of IL-35 is closely related to the development of lung cancer. Among all the results of the GSEA analysis, we screened two pathways with high enrichment scores, tight junction pathway and the NSCLC pathway. While the target gene enrichment analysis of Dendrobium houshanense in Fig. 2A was also in tight junction and multiple tumor regulatory pathways, we concluded that Dendrobium houshanense is likely to be regulated in NSCLC through IL-35 mediated signaling pathway regulation in the development of NSCLC as well as anti-tumor immune effects.

### Dendrobium huoshanense inhibits proliferation, migration and invasion of NSCLC through the FXR1-IL-35-ACTR3 signaling pathway

**Fig. 4:**
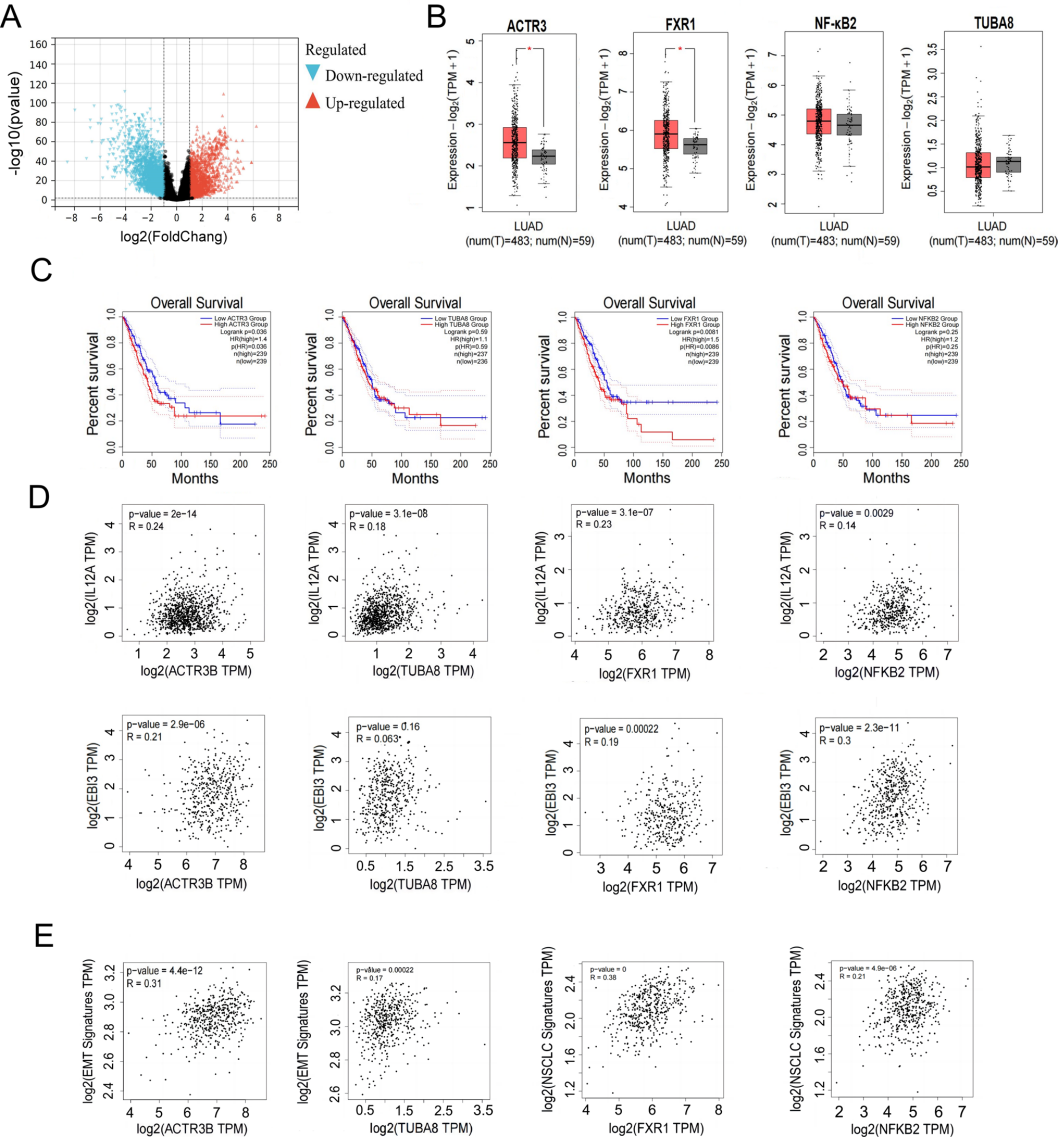
Dendrobium huoshanense inhibits proliferation, migration and invasion of NSCLC through the FXR1-IL-35-ACTR3 signaling pathway. **A** Volcano map analysis of differentially regulated genes in LUAD. **B** The mRNA Expression of ACTR3, FXR1, NF-κB, TUBA8 in LUAD. **C** The Gehan-Breslow–Wilcoxon test showed that the protein expression of ACTR3, FXR1 predicted poor survival in LUAD patients, and TUBA8, NF-κB2 did not correlate with patient survival. **D** Correlations between ACTR3, FXR1, NF-κB2, TUBA8 and IL-12A, EBI3 mRNA levels, ACTR3, FXR1 and IL-12A, EBI3 mRNA levels are positively correlated, one point represents one sample. **E** Correlations between ACTR3, TUBA8 and EMT signatures, FXR1, NF-κB and NSCLC signatures

As shown in Fig. 4A, the red dots on the right represent genes that are highly expressed compared to normal individuals, while the blue dots on the left are genes that are lowly expressed in LUAD patients. Through previous KEGG enrichment analysis of Dendrobium houshanense target genes and GSEA enrichment analysis of IL-35, we screened four overlapping genes, FXR1, NF-κB2, ACTR3, and TUBA8, in their common pathways, tight junction pathway and NSCLC pathway. Tight junction pathway and NSCLC pathway. FXR1 and ACTR3 were highly expressed in LUAD, while NF-κB2 and TUBA8 were not significantly differentially expressed in LUAD (Fig. 4B). As FXR1, NF-κB2, ACTR3, and TUBA8 have been shown to be biologically important, we sought to investigate their clinical value. We next did a survival analysis of the four genes mentioned above and found that high expression of FXR1 or ACTR3 was significantly associated with poor patient survival and a poor prognosis, while high expression of NF-κB2 or TUBA8 was not significantly associated with patients and their overall survival (no value for patient prognosis) (Fig. 4C). Analysis showed that FXR1 and ACTR3 were significantly positively correlated with IL-35 (*R = *0.23, *R = *0.24), while NF-κB2 and TUBA8 were not significantly correlated with IL-35 compared to FXR1 and ACTR3 (Fig. 4D), This led us to conclude that FXR1 and ACTR3 are significantly associated with IL-35, have prognostic value, and may be important targets for the action of Dendrobium houshanense. It has been shown that the tight junction pathway plays an important role in the tumor microenvironment and is closely related to cancer migration and invasion [15]. We further investigated the effect of high expression of IL-35-related FXR1 and ACTR3 on tumor microenvironment (TME) and found that ACTR3 was significantly associated with EMT signatures and FXR1 was positively associated with NSCLC signatures, which were significantly and positively correlated (Fig. 4E). In summary, we suggest that Dendrobium houshanense are likely to directly regulate NSCLC proliferation through FXR1, regulate the tumor microenvironment through FXR1-IL-35-ACTR3 axis, and influence tumor cell migration and invasion.

### cDHPs inhibit the development of NSCLC in vivo by suppressing IL-35 expression

**Fig. 5:**
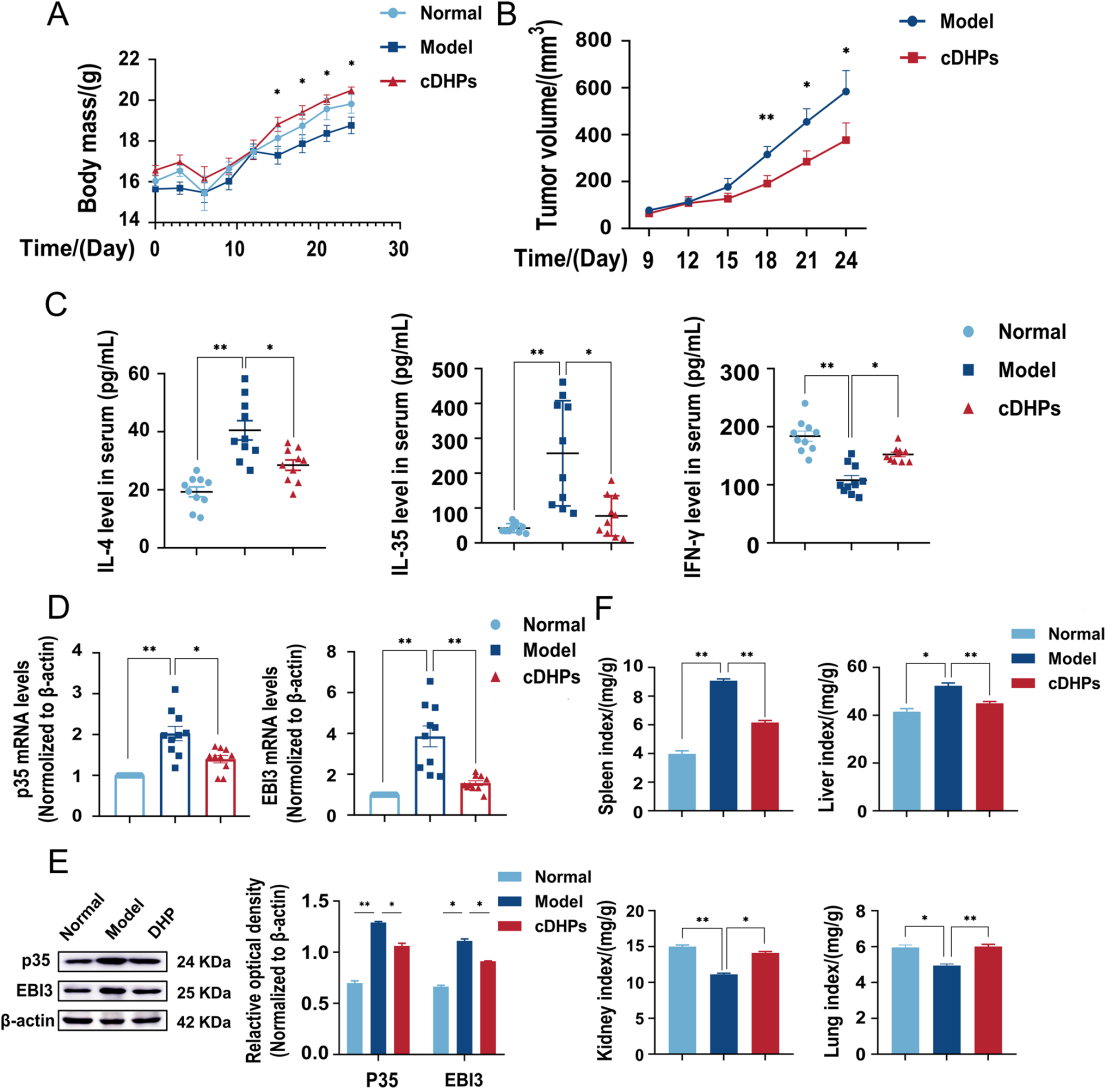
cDHPs inhibit the development of NSCLC in vivo by suppressing IL-35 expression. **A** Curve of mice body mass. **B** Tumor volume curve. **C** Measurement of IL-35, IL-4, IFN-γ concentrations after treatment with cDHPs by ELISA. **D**, **E** After cDHPs treatment, p35, EBI3 protein levels and mRNA levels were detected by Western blot and RT-PCR, respectively. **F** Organ index for Spleen, liver, kidney and lung. *p < 0.05; ∗∗*p* < 0.01

To test the previous conjecture, we conducted animal experiments. To construct a lung cancer tumor-bearing mouse model, we injected 1 × 10^6^ LLC into the right axilla of C57BL/6 mice subcutaneously, and after tumor formation, thirty mice were randomly divided into three groups of 10 each: the normal group, the model group, and the cDHPs group. In this study, cDHPs 200 mg/kg was administered orally by gavage based on the initial concentrations explored in the pre-experiment and the effective dosage conversions in humans and mice. It is possible to notice that from the 12th day of the experiment, the body weight of the animals that had the tumor-induced was lower than that of the animals in the control group. However, the animals that had the tumor-induced and were treated with the cDHPs gradually regained weight (Fig. 5A). Meanwhile, the volume of tumors was significantly suppressed after treatment (Fig. 5B). To further explore the effects of cDHPs on the immune microenvironment. We measured serum levels of IL-4, IL-35, and IFN-γ by ELISA after cDHPs treatment, and IL-35 and IL-4 levels decreased while IFN-γ levels increased (Fig. 5C). The mRNA levels of p35 and EBI3 were decreased in the model group by RT-PCR and protein levels by Western blotting, and their protein levels were similarly decreased and recovered after cDHPs treatment, suggesting that cDHPs may have inhibited gene expression by suppressing the transcription of IL-35 (Fig. 5D, E). At the same time, organ analysis of the mice showed that the spleen and liver of the model mice were significantly larger compared to the normal group, indicating that the spleen had become congested and enlarged after tumor formation, which may have reduced the normal immune function. cDHPs treatment decreased the spleen index and liver index, and the spleen was restored. The lungs and kidneys of the tumor-forming model group were compressed and reduced, and the organ indices decreased. cDHPs treatment increased the lung and kidney indices, indicating that cDHPs could protect the organs of tumor-bearing mice from tumors (Fig. 5F) (Table 2).

**Table 2:** Organ indexes of spleen, liver, kidney and lung

mg/g	Normal	Model	cDHPs
Spleen	4.0±0.7	9.1±0.4**	6.2±0.5**
Lung	6.0±0.4	4.9±0.3*	6.0±0.4**
Kidney	15.0±0.7	11.1±0.5**	14.1±0.6*
Liver	41.5±4.1	52.3±3.8*	45.0±2.6**

*p < 0.05; **p < 0.01

### cDHPs inhibit the conversion of iTr35 by inhibiting IL-35 to prevent tumor immune evasion

**Fig. 6:**
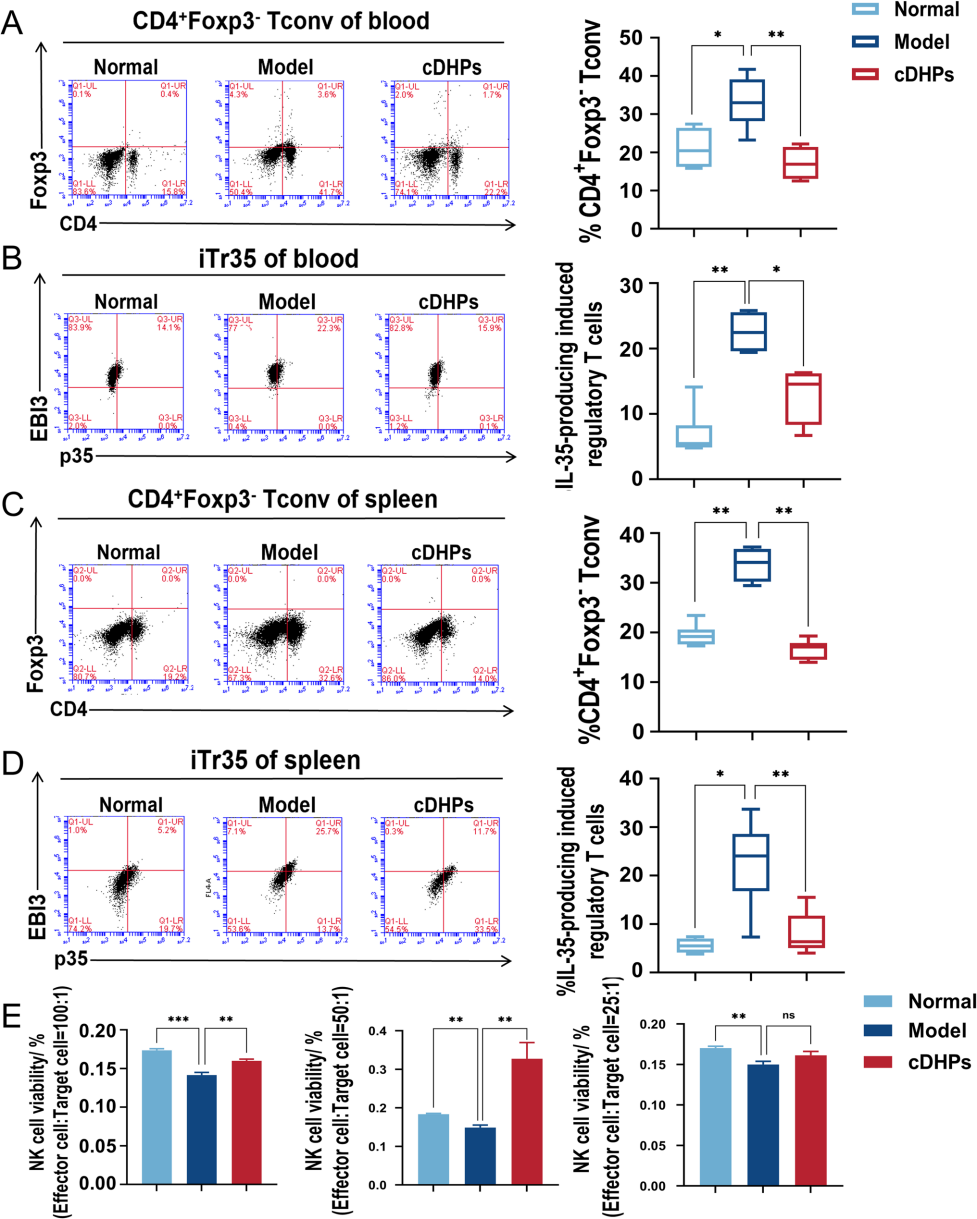
cDHPs inhibit the conversion of iTr35 by inhibiting IL-35 to prevent tumor immune evasion. **A**, **B** Detection of CD4^+^Foxp3^−^ Tconv and iTr35 levels in blood after treatment with cDHPs by flow cytometry, respectively. **C**, **D** Detection of CD4^+^Foxp3^−^ Tconv and iTr35 levels in spleen and after treatment with cDHPs by flow cytometry, respectively. E: Effects of dendrobium huoshanense polysaccharides on NK cell viability. *p < 0.05; ∗∗*p* < 0.01

We hypothesized that cDHPs could inhibit the conversion of naive T cells and Tregs into iTr35. We examined CD4^+^Foxp3^−^ Tconv and iTr35 in the spleen and blood by flow cytometry after sorting, CD4^+^Foxp3^−^ Tconv expression was increased in tumor-bearing mice (Fig. 6A, C). The conversion of iTr35 was inhibited after treatment with cDHPs, as shown in Fig. 6B, D. This is also consistent with previous single-cell sequencing analyses.

### cDHPs activate the innate immune system to kill tumor cells by enhancing NK cell activity

NK cells were sorted in the spleen at 100:1, 50:1, and 25:1 for target cells (YAC-1) and effector cells (NK cells), respectively, and the activity of NK cells was measured when effector cells: target cells = 50:1. The most significant increase in NK cell activity by cDHPs treatment was observed when the effector cells: target cells ratio was 50:1, and there was no effect on NK cells by treatment when the effector cells: target cells ratio was 25:1 (Fig. 6E). Therefore, cDHPs can promote the increase of NK cell activity in the body and induce the activation of anti-tumor immunity.

### Combination of cDHPs improves the efficacy of PD-1/PD-L1 antibodies for LUAD patients

**Fig. 7:**
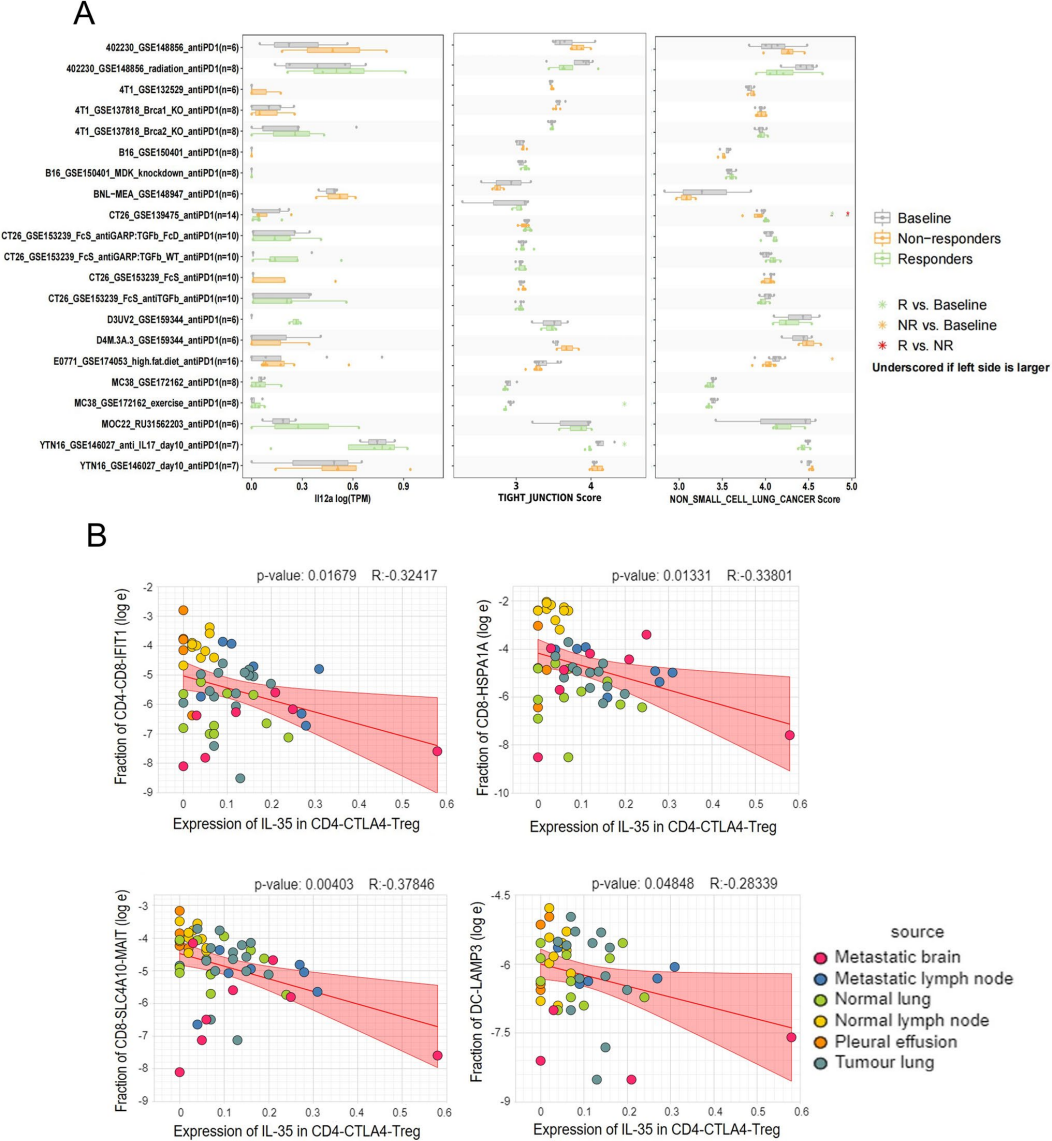
cDHPs activate the innate immune system to kill tumor cells by enhancing NK cell activity. **A** IL12A expression level, tight junction score and NSCLC score of baseline, non-responders and responders in homologous mouse immunotherapy cohorts. **B** Expression and cell composition correlation between IL-35 expression in CD4-CTLA4-Treg and composition of CD4-CD8-IFTH1, CD8-HSP1A1, CD8-SLC4A10 and DC-LAMP3 cells

Immunotherapy based on immune checkpoint inhibitors has recently shown great promise in a variety of tumors [16]. To investigate the effect of the combination of Dendrobium houshanense and anti-PD-1/PD-L1 on the efficacy of ICIs alone, we obtained sequencing data from TISMO in the database for analysis of the immunotherapy cohort of hormonal mice, before and after treatment with PD-1/PD-L1 antibodies, respectively. We found that IL-12A expression was higher in tumor tissues of mice receiving PD-1/PD-L1 antibodies than in baseline data (Fig. 7A) and that the tight junction and NSCLC pathway were also activated in non-responders. Also, analysis of single-cell sequencing data showed that Treg could induce a reduction in multiple immune effector cells via IL-35 (Fig. 7B). We thus hypothesized that cDHPs could improve PD-1/PD-L1 antibody efficacy by increasing effector T cell and NK cell infiltration abundance through inhibition of IL-35 expression and enhancing anti-tumor immunity (Supplementary material 2).

## Discussion

Lung cancer is among the most prevalent malignancies globally and continues to have a significant mortality burden [1]. Advanced non-small cell lung cancer (NSCLC) poses considerable therapeutic challenges, primarily due to the limitations of traditional chemotherapy, frequent metastases at initial diagnosis, and a high likelihood of post-treatment recurrence [17]. Recent advancements in cancer immunotherapy, particularly immune checkpoint inhibitors (ICIs), have shown promise as innovative anti-tumor strategies. ICIs are now being increasingly explored as potential therapeutic options for early-stage NSCLC, with growing evidence supporting their clinical relevance [18–20].

In recent years, a large number of basic studies and clinical trials have shown that polysaccharide components in natural products have attracted widespread attention because of their antioxidant, anti-inflammatory, and anti-tumor effects [21]. cDHPs exhibit a potential anticancer activity mainly through the activation of immune cell and modulation of signaling pathways related to growth, proliferation, migration, invasion and activation of apoptotic mechanisms. In this study, we confirmed the anti-tumor effect of cDHPs treatment on LUAD mice. Then we found it targets tumor through the FXR1-IL-35 axis and causes CD4^+^ T cells into IL-35-producing induced regulatory T cells (iTr35) to activate anti-tumor immunity to enhance the anti-tumor ability.

With the development of network pharmacology, bioinformatics, and single-cell sequencing technology, new approaches are available for researchers to unravel the therapeutic mechanisms of tumor from massive amounts of histologic data. Therefore, in this study, we first analyzed the active ingredients and therapeutic targets of Dendrobium huoshanense in detail using network pharmacology and screened out 8 active ingredients and 159 active targets. To explore the mechanism of action of Dendrobium huoshanense and the biological functions of its target genes, a constituent-target network was constructed. Then, the biological functions of the therapeutic targets were interpreted by KEGG, Hallmark, and GO enrichment analysis, and the targets were mainly enriched in pathogenic escherichia coli infection, phagosome, gap junction, tight junction, apoptosis, one carbon pool by folate, colorectal cancer and other pathways, suggesting that cDHPs may inhibit the development of tumor and activate anti-tumor immunity through various pathways. To investigate the reprogramming mechanism of cDHPs on the microenvironment of NSCLC and the interaction with IL-35, we combined multi-omics and single-cell sequencing data analysis and found that high expression of IL-35 in tumor tissues of LUAD patients in the TCGA cohort, as well as the amplification of the gene encoding IL-35, were significantly associated with reduced infiltration abundance of CD8^+^ T cells, DC cells, and neutrophils in TME. The mechanism by which IL-35 prevents immune evasion by inducing Treg cell proliferation and suppressing CD8^+^ T cells was discovered. Single gene GSEA analysis of IL-35 led us to note that Dendrobium houshanense likely regulates an immunosuppressive cytokine, IL-35, through the tight junction pathway, thereby improving the immunosuppressive tumor microenvironment and preventing immune evasion from tumors. The tight junction pathway is associated with cell-to-cell junctions, and epithelial and endothelial cells act as sentinels in most living systems act as sentinels, providing a protective barrier for various organs from the surrounding environment and helping to maintain dynamic homeostasis in the body's internal environment [22]. This plays a key role in the epithelial-mesenchymal transition of tumors and tumor metastasis. This suggests that cDHPs targeting the tight junction pathway have a dual anti-tumor effect by blocking tumor EMT and immune evasion. In the TCGA cohort, FXR1 and ACTR3, key genes in the tight junction and NSCLC pathways targeted by cDHPs were significantly positively associated with IL-35, and FXR1 was positively associated with key driver genes for NSCLC development, while ACTR3 was positively associated with EMT-related genes. FXR1 is an RNA-binding protein FXR1 is an RNA-binding protein that plays an important role in differentiation, development, and tumorigenesis [23] and has been found to regulate the expression levels of many genes, including increasing the expression levels of specific cytokines and chemokines associated with the induction of cell migration, and influencing cell growth and cancer by controlling cell cycle and growth factor gene expression [24]. The immunosuppressive properties of IL-35 are an important barrier to immunotherapy in solid tumors. Important barrier to immunotherapy in solid tumors, where cells and cytokines in this tumor microenvironment severely impede the infiltration and proliferation of immune cells and reduce the anti-cancer activity of tumor-infiltrating immune cells (which are closely associated with tumor proliferation, angiogenesis, and metastasis) [25, 26]. In many solid cancers, elevated IL-35 is strongly associated with poor cancer prognosis. In CD4^+^ T cells, it inhibits the Th1 and Th17 cell responses, enhances Treg responses, and reduces the cytotoxic effects of cytokines produced by CD4^+^ T cells in NSCLC. In Tconv cells, BCC-derived IL-35 increased the secretion of the suppressive cytokine IL-10 while decreasing the secretion of the Th1-type cytokine IFN-γ and the Th17-type cytokine IL-17 [27]. Yong Gang et al. found through their study that p35 expression was also associated with the spread of gastric cancer cells, and that EBI3 was expressed at significantly higher levels in gastric cancer cells than in normal gastric mucosal cells [28]. Huizhi Sun et al. found that tumor-derived IL-35 mediates the accelerated resistance of PDAC to gemcitabine (GEM). Additionally, they discovered that GEM-resistant cells have significantly higher levels of IL-35 expression. Mechanistically, aberrantly expressed IL-35 triggers transcriptional activation of SOD2 expression via GP130-STAT1 signaling, scavenging reactive oxygen species (ROS) and leading to GEM resistance [29]. In hepatocellular carcinoma, M1 macrophages secreted significantly higher levels of IL-35 than M2 macrophages, which promoted the EMT process by activating STAT3 in cancer cells, and cancer cell-tumor microenvironment interactions could promote tumor growth and metastasis [30]. This suggests to us that cDHPs most likely inhibit the FXR1-IL-35-ACTR3 axis to block tumor proliferation, immune evasion, and metastasis.

We verified the specific mechanism of cDHPs’ anti-tumor effects through animal experiments. cDHPs could inhibit the growth of LLC transplantation tumor and protect the basic organ functions to some extent. cDHPs treatment decreased IL-35 and IL-4 levels while increasing IFN-γ levels in serum and spleen. Studies have shown that IL-4 has a stimulatory effect on proliferation and survival in pancreatic cancer cells acting as an autocrine growth factor, and cancer-derived IL-4 may also inhibit cancer-directed immune surveillance in vivo, thereby promoting pancreatic tumor growth and metastasis [31]. IFN-γ is the most important cytokine involved in anti-tumor immunity. Mice lacking the IFN-γ receptor and STAT1 have a significant increase in chemical tumor growth after exposure to chemical carcinogens, demonstrating IFN-γ's anti-cancer ability. Patients whose tumors express both IFN-γ and PD-L1 may be a better predictor of PD-1/PD-L1 blockade immunotherapy [32]. p35, EBI3 at the mRNA level the protein level was decreased by cDHPs treatment, revealing that cDHPs may be affecting IL-35 expression at the translational level. CD4^+^ T cells in the blood and spleen of mice recovered after cDHPs administration and CD4^+^ T cells in the tumor microenvironment were converted to suppressive iTr35 cells by the FXR1-IL-35 axis, while NK cells showed increased viability after treatment, confirming the previous conjecture that the anti-tumor immune effect of the organism was enhanced. Finally, our analysis using sequencing data from a homologous mouse immunotherapy cohort revealed high IL-35 expression as well as the core pathway Tight junction targeted by Dendrobium houshanense, activation of NSCLC against the negative effects of ICIs treatment such as PD-1/PD-L1, and anti-CTLA-4. It provides initial validation for combined cDHPs and ICI therapy and new ideas to break through the bottleneck currently faced by ICI therapy.

In summary, we have identified a significant inhibitory effect of cDHPs on the development of lung cancer. However, elucidated the crucial role by the FXR1-IL-35-ACTR3 axis in mediating the anti-tumor and immune regulatory effects. However, the relationship of FXR1-IL-35-ACTR3 axis the relationship of in the current experiments needs to be validated by in-depth molecular biology experiments, and the combination of cDHPs with PD-1/PD-L1 needs to continue to be validated by animal experiments, contributing to a more profound understanding of the application of herbal anti-tumor immunotherapy, which will also be our next step.

## Conclusion

Our study reveals that cDHPs suppress IL-35 expression and block the conversion of CD4^+^ T cells to iTr35 through the FXR1-IL-35 axis to inhibit NSCLC development. Combination of cDHPs improves the efficacy of PD-1/PD-L1 antibodies for LUAD patients. It provides a preclinical basis for the use of cDHPs in the treatment of lung cancer and provides new insights into the FXR1-IL-35 axis signaling pathway as an anti-NSCLC target.

## Supplementary Information

Below is the link to the electronic supplementary material.

Supplementary file1 (PDF 310 KB)

Supplementary file2 (PDF 51 KB)

Supplementary file3 (DOCX 22 KB)

## Data Availability

The datasets used during the current study are available from the corresponding author on reasonable request. We can provide raw data and code if it is a reasonable request.

## Abbreviations

cDHPs: Dendrobium huoshanense polysaccharide
NSCLC: Non-small cell lung cancer
PD-1: Programmed cell death-1
PD-L1: Programmed death-ligand 1
Tregs: T regulatory cells
FXR1: Fragile X-related protein-1
LLC: Lewis lung carcinoma
LUAD: Lung adenocarcinoma
LUSC: Lung squamous cell carcinoma
iTr35: IL-35-producing induced regulatory T cells
GO: Gene Ontology
KEGG: Kyoto Encyclopedia of Genes and Genomes
