# Supplementary figures and images for: Dendrobium huoshanense polysaccharide inhibits NSCLC proliferation and immune evasion via FXR1-IL-35 axis signaling pathway
Source: J Nat Med. 2025 Apr 21;79(4):863–78. doi: 10.1007/s11418-025-01894-7 (PMC12228671; doi:10.1007/s11418-025-01894-7)

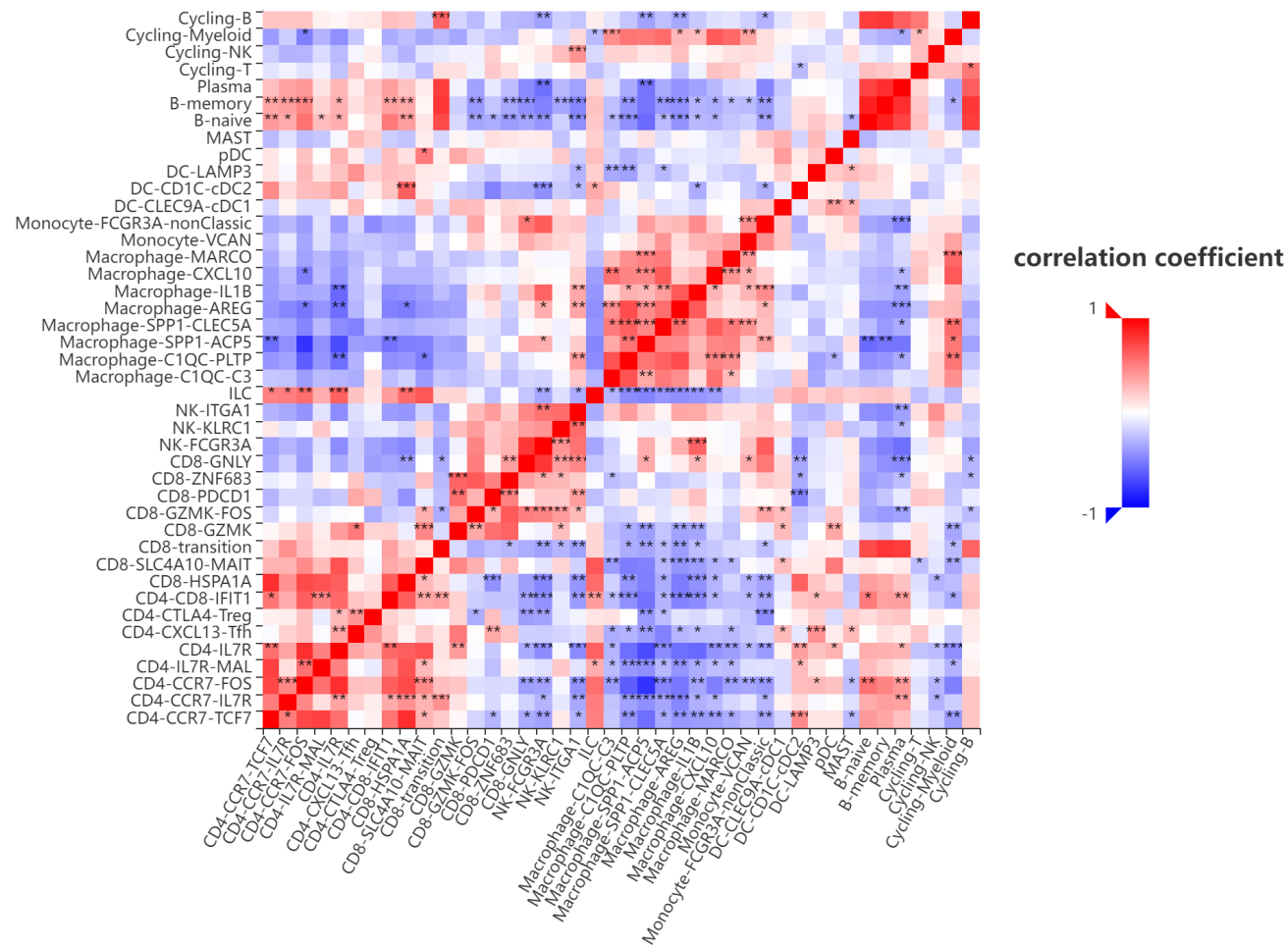

Supplement: Supplementary file 1 — Supplementary file1 (PDF 310 KB) [file 11418_2025_1894_MOESM1_ESM.pdf]
