# Supplementary material for: Dendrobium huoshanense polysaccharide inhibits NSCLC proliferation and immune evasion via FXR1-IL-35 axis signaling pathway
Source: J Nat Med. 2025 Apr 21;79(4):863–78. doi: 10.1007/s11418-025-01894-7 (PMC12228671; doi:10.1007/s11418-025-01894-7)
Supplement: Supplementary file 2 — Supplementary file2 (PDF 51 KB) [file 11418_2025_1894_MOESM2_ESM.pdf]

Ingredient	Gene ID
Daucosterol	AR
Daucosterol	PGR
Daucosterol	ESR1
Daucosterol	NCOA2
Scoparone	ADRB2
Scoparone	CHRM1
Scoparone	DCT
Scoparone	DECR1
Scoparone	GRIA2
Scoparone	CXCL8
Scoparone	MAOB
Scoparone	MTRR
Scoparone	NFKBIA
Scoparone	NOS1
Scoparone	NOS2
Scoparone	NOS3
Scoparone	POR
Scoparone	PTGS1
Scoparone	PTGS2
Scoparone	RELA
Scoparone	CCL2
Scoparone	SLC6A4
Scoparone	TYR
Scoparone	TYRP1
Scoparone	NDOR1
Scoparone	TYW1
Scoparone	PIM1
Scoparone	ESR1
Scoparone	CDK2
Scoparone	DPEP1
Scoparone	GABRA1
Scoparone	LTA4H
Scoparone	PPARG
Scoparone	DPP4
Scoparone	CCNA2
Scoparone	PKIA
Scoparone	CA2
Rutaecarpine	AR
Rutaecarpine	CYP1A2
Rutaecarpine	CYP2B6
Rutaecarpine	CYP3A4
Rutaecarpine	HTR3A
Rutaecarpine	IL4

Rutaecarpine	MMP2
Rutaecarpine	MMP9
Rutaecarpine	PTGS1
Rutaecarpine	PTGS2
Rutaecarpine	RXRA
Rutaecarpine	SCN5A
Rutaecarpine	TNF
Rutaecarpine	CA2
Rutaecarpine	CCNA2
Rutaecarpine	ESR1
Rutaecarpine	CDK2
Rutaecarpine	ESR2
Rutaecarpine	PIM1
Rutaecarpine	PRSS1
Rutaecarpine	DPP4
Rutaecarpine	CHEK1
Rutaecarpine	ACHE
Rutaecarpine	MAPK14
Rutaecarpine	PPARG
Rutaecarpine	NOS2
Rutaecarpine	GSK3B
Nodakenetin	ADRB2
Nodakenetin	CCNA2
Nodakenetin	CDK2
Nodakenetin	CHRM1
Nodakenetin	ESR1
Nodakenetin	MAOB
Nodakenetin	PTGS1
Nodakenetin	PTGS2
Nodakenetin	RXRA
Nodakenetin	SLC6A2
Nodakenetin	SLC6A4
Nodakenetin	DPP4
Nodakenetin	CHRM2
Nodakenetin	ADRA2B
Nodakenetin	CHEK1
Nodakenetin	NOS2
Nodakenetin	PKIA
Nodakenetin	PDE3A
Nodakenetin	LTA4H
Nodakenetin	DPEP1
Nodakenetin	CA2
Panaxadiol	AR
Panaxadiol	MMP9

Panaxadiol	NR3C1
Evodiamine	IL6
Evodiamine	STAT3
Evodiamine	JAK2
Evodiamine	MAPK3
Evodiamine	MAPK1
Evodiamine	SRC
Evodiamine	TRPV1
Polysaccharides	ACTA1
Polysaccharides	ACTA2
Polysaccharides	ACTB
Polysaccharides	ACTC1
Polysaccharides	ACTG1
Polysaccharides	ACTG2
Polysaccharides	ACTL6A
Polysaccharides	AKT1
Polysaccharides	RHOA
Polysaccharides	RHOB
Polysaccharides	RHOC
Polysaccharides	BDNF
Polysaccharides	FMR1
Polysaccharides	FOS
Polysaccharides	G6PD
Polysaccharides	GCG
Polysaccharides	GPLD1
Polysaccharides	KCNA5
Polysaccharides	NCL
Polysaccharides	SLC26A4
Polysaccharides	PLD1
Polysaccharides	PLD2
Polysaccharides	SLC6A3
Polysaccharides	SLC12A1
Polysaccharides	SLC12A2
Polysaccharides	SLC12A4
Polysaccharides	SNAP25
Polysaccharides	SNCA
Polysaccharides	VEGFA
Polysaccharides	FXR1
Polysaccharides	SNAP23
Polysaccharides	SNAP29
Polysaccharides	FXR2
Polysaccharides	H6PD
Polysaccharides	SLC12A6
Polysaccharides	ACTR3

Polysaccharides	ACTR2
Polysaccharides	ACTR1B
Polysaccharides	ACTR1A
Polysaccharides	SLC12A7
Polysaccharides	ACTL7B
Polysaccharides	ACTL7A
Polysaccharides	WDTC1
Polysaccharides	DICER1
Polysaccharides	TARDBP
Polysaccharides	DDX58
Polysaccharides	PLD3
Polysaccharides	DROSHA
Polysaccharides	ACTL6B
Polysaccharides	FAM3B
Polysaccharides	ACTR10
Polysaccharides	ACTR3B
Polysaccharides	SLC12A5
Polysaccharides	FANCM
Polysaccharides	IFIH1
Polysaccharides	DHX58
Polysaccharides	ACTL8
Polysaccharides	ACTRT3
Polysaccharides	SMIM3
Polysaccharides	ACTR8
Polysaccharides	KRT71
Polysaccharides	SNAP47
Polysaccharides	KRT74
Polysaccharides	ACTRT1
Polysaccharides	ACTRT2
Polysaccharides	KRT72
Polysaccharides	SIK1
Polysaccharides	ACTL10
Polysaccharides	ACTL9
Polysaccharides	KRT73
Polysaccharides	ACTBL2
Polysaccharides	POTEE
Polysaccharides	POTEI
Polysaccharides	POTEJ
Polysaccharides	ACTR3C
Erianin	TUBA4A
Erianin	TUBA3C
Erianin	TUBB2A
Erianin	TUBG1
Erianin	TUBA1A

Erianin	TUBA1B
Erianin	TUBB3
Erianin	TUBB4A
Erianin	TUBB4B
Erianin	TUBG2
Erianin	TUBD1
Erianin	TUBE1
Erianin	TUBA8
Erianin	TUBAL3
Erianin	TUBB1
Erianin	TUBB6
Erianin	TUBA1C
Erianin	TUBA3E
Erianin	TUBB
Erianin	TUBB2B
