# Supplementary material for: Dendrobium huoshanense polysaccharide inhibits NSCLC proliferation and immune evasion via FXR1-IL-35 axis signaling pathway
Source: J Nat Med. 2025 Apr 21;79(4):863–78. doi: 10.1007/s11418-025-01894-7 (PMC12228671; doi:10.1007/s11418-025-01894-7)
Supplement: Supplementary file 3 — Supplementary file3 (DOCX 22 KB) [file 11418_2025_1894_MOESM3_ESM.docx]

| **Ingredient** | **Gene ID** |
| --- | --- |
| Daucosterol | AR |
| Daucosterol | PGR |
| Daucosterol | ESR1 |
| Daucosterol | NCOA2 |
| Scoparone | ADRB2 |
| Scoparone | CHRM1 |
| Scoparone | DCT |
| Scoparone | DECR1 |
| Scoparone | GRIA2 |
| Scoparone | CXCL8 |
| Scoparone | MAOB |
| Scoparone | MTRR |
| Scoparone | NFKBIA |
| Scoparone | NOS1 |
| Scoparone | NOS2 |
| Scoparone | NOS3 |
| Scoparone | POR |
| Scoparone | PTGS1 |
| Scoparone | PTGS2 |
| Scoparone | RELA |
| Scoparone | CCL2 |
| Scoparone | SLC6A4 |
| Scoparone | TYR |
| Scoparone | TYRP1 |
| Scoparone | NDOR1 |
| Scoparone | TYW1 |
| Scoparone | PIM1 |
| Scoparone | ESR1 |
| Scoparone | CDK2 |
| Scoparone | DPEP1 |
| Scoparone | GABRA1 |
| Scoparone | LTA4H |
| Scoparone | PPARG |
| Scoparone | DPP4 |
| Scoparone | CCNA2 |
| Scoparone | PKIA |
| Scoparone | CA2 |
| Rutaecarpine | AR |
| Rutaecarpine | CYP1A2 |
| Rutaecarpine | CYP2B6 |
| Rutaecarpine | CYP3A4 |
| Rutaecarpine | HTR3A |
| Rutaecarpine | IL4 |
| Rutaecarpine | MMP2 |
| Rutaecarpine | MMP9 |
| Rutaecarpine | PTGS1 |
| Rutaecarpine | PTGS2 |
| Rutaecarpine | RXRA |
| Rutaecarpine | SCN5A |
| Rutaecarpine | TNF |
| Rutaecarpine | CA2 |
| Rutaecarpine | CCNA2 |
| Rutaecarpine | ESR1 |
| Rutaecarpine | CDK2 |
| Rutaecarpine | ESR2 |
| Rutaecarpine | PIM1 |
| Rutaecarpine | PRSS1 |
| Rutaecarpine | DPP4 |
| Rutaecarpine | CHEK1 |
| Rutaecarpine | ACHE |
| Rutaecarpine | MAPK14 |
| Rutaecarpine | PPARG |
| Rutaecarpine | NOS2 |
| Rutaecarpine | GSK3B |
| Nodakenetin | ADRB2 |
| Nodakenetin | CCNA2 |
| Nodakenetin | CDK2 |
| Nodakenetin | CHRM1 |
| Nodakenetin | ESR1 |
| Nodakenetin | MAOB |
| Nodakenetin | PTGS1 |
| Nodakenetin | PTGS2 |
| Nodakenetin | RXRA |
| Nodakenetin | SLC6A2 |
| Nodakenetin | SLC6A4 |
| Nodakenetin | DPP4 |
| Nodakenetin | CHRM2 |
| Nodakenetin | ADRA2B |
| Nodakenetin | CHEK1 |
| Nodakenetin | NOS2 |
| Nodakenetin | PKIA |
| Nodakenetin | PDE3A |
| Nodakenetin | LTA4H |
| Nodakenetin | DPEP1 |
| Nodakenetin | CA2 |
| Panaxadiol | AR |
| Panaxadiol | MMP9 |
| Panaxadiol | NR3C1 |
| Evodiamine | IL6 |
| Evodiamine | STAT3 |
| Evodiamine | JAK2 |
| Evodiamine | MAPK3 |
| Evodiamine | MAPK1 |
| Evodiamine | SRC |
| Evodiamine | TRPV1 |
| Polysaccharides | ACTA1 |
| Polysaccharides | ACTA2 |
| Polysaccharides | ACTB |
| Polysaccharides | ACTC1 |
| Polysaccharides | ACTG1 |
| Polysaccharides | ACTG2 |
| Polysaccharides | ACTL6A |
| Polysaccharides | AKT1 |
| Polysaccharides | RHOA |
| Polysaccharides | RHOB |
| Polysaccharides | RHOC |
| Polysaccharides | BDNF |
| Polysaccharides | FMR1 |
| Polysaccharides | FOS |
| Polysaccharides | G6PD |
| Polysaccharides | GCG |
| Polysaccharides | GPLD1 |
| Polysaccharides | KCNA5 |
| Polysaccharides | NCL |
| Polysaccharides | SLC26A4 |
| Polysaccharides | PLD1 |
| Polysaccharides | PLD2 |
| Polysaccharides | SLC6A3 |
| Polysaccharides | SLC12A1 |
| Polysaccharides | SLC12A2 |
| Polysaccharides | SLC12A4 |
| Polysaccharides | SNAP25 |
| Polysaccharides | SNCA |
| Polysaccharides | VEGFA |
| Polysaccharides | FXR1 |
| Polysaccharides | SNAP23 |
| Polysaccharides | SNAP29 |
| Polysaccharides | FXR2 |
| Polysaccharides | H6PD |
| Polysaccharides | SLC12A6 |
| Polysaccharides | ACTR3 |
| Polysaccharides | ACTR2 |
| Polysaccharides | ACTR1B |
| Polysaccharides | ACTR1A |
| Polysaccharides | SLC12A7 |
| Polysaccharides | ACTL7B |
| Polysaccharides | ACTL7A |
| Polysaccharides | WDTC1 |
| Polysaccharides | DICER1 |
| Polysaccharides | TARDBP |
| Polysaccharides | DDX58 |
| Polysaccharides | PLD3 |
| Polysaccharides | DROSHA |
| Polysaccharides | ACTL6B |
| Polysaccharides | FAM3B |
| Polysaccharides | ACTR10 |
| Polysaccharides | ACTR3B |
| Polysaccharides | SLC12A5 |
| Polysaccharides | FANCM |
| Polysaccharides | IFIH1 |
| Polysaccharides | DHX58 |
| Polysaccharides | ACTL8 |
| Polysaccharides | ACTRT3 |
| Polysaccharides | SMIM3 |
| Polysaccharides | ACTR8 |
| Polysaccharides | KRT71 |
| Polysaccharides | SNAP47 |
| Polysaccharides | KRT74 |
| Polysaccharides | ACTRT1 |
| Polysaccharides | ACTRT2 |
| Polysaccharides | KRT72 |
| Polysaccharides | SIK1 |
| Polysaccharides | ACTL10 |
| Polysaccharides | ACTL9 |
| Polysaccharides | KRT73 |
| Polysaccharides | ACTBL2 |
| Polysaccharides | POTEE |
| Polysaccharides | POTEI |
| Polysaccharides | POTEJ |
| Polysaccharides | ACTR3C |
| Erianin | TUBA4A |
| Erianin | TUBA3C |
| Erianin | TUBB2A |
| Erianin | TUBG1 |
| Erianin | TUBA1A |
| Erianin | TUBA1B |
| Erianin | TUBB3 |
| Erianin | TUBB4A |
| Erianin | TUBB4B |
| Erianin | TUBG2 |
| Erianin | TUBD1 |
| Erianin | TUBE1 |
| Erianin | TUBA8 |
| Erianin | TUBAL3 |
| Erianin | TUBB1 |
| Erianin | TUBB6 |
| Erianin | TUBA1C |
| Erianin | TUBA3E |
| Erianin | TUBB |
| Erianin | TUBB2B |
